# Recognition for *Vision*

**DOI:** 10.3390/vision3040069

**Published:** 2019-12-17

**Authors:** Andrew J. Parker

**Affiliations:** Department of Physiology, Anatomy and Genetics, University of Oxford, Oxford OX1 3PT, UK; andrew.parker@dpag.ox.ac.uk

When children grow up, the first word or first step in walking is always a significant event. These events rarely coincide neatly with calendar dates. So it is with the birth and growth of a new journal. *Vision* suddenly has a new set of identities.

The main open source for paper-scanning and literature searches for current and future readers of this journal would most likely be PubMed. This database is well known as the standard open-source database in medical sciences. We are very pleased to be able to announce that *Vision* can now be found on PubMed at https://www.ncbi.nlm.nih.gov/pmc/journals/3742/. This is a major step forward for the new journal in expanding its visibility and its credibility as a permanent new feature in the research space.

The indexing on PubMed will include all the papers published to date, thus increasing their visibility. We will also now be present on Scopus, as of October 2019, and are pleased to have this recognition. Both of these advances are backed by the policy of our publisher, MDPI, who have arranged for all of the articles published in their journals to be deposited in the Swiss National Library electronic archiving system. These archives would survive any change of ownership, and this arrangement effectively ensures that all papers published in *Vision* remain in the public domain in perpetuity.

We are very grateful for the efforts of the current editors and referees in helping us get to this new status. Nonetheless, we can only be a viable journal if research groups send their papers forward. For those who have been involved in the early stages of this new journal, we believe that the current news will be especially welcome.

I would also like to draw your attention to some other, recent activity of our publishers, MDPI. They have been a major sponsor of the *First Basel Sustainable Publishing Forum*. This is an important effort for the future of scientific publishing. We wish our umbrella organization well in taking forward these efforts and encourage them to go further.

An additional scientific development that we greatly encourage at the moment is further collections of Special Issues, that is to say focused collections of papers covering specific topics in visual science. These Special Issues provide a common reference point for researchers specializing in those topics and provide new generations of students and researchers with a compact way of accessing particular research themes. Thanks to electronic publishing, the size and format of Special Issues have become much more flexible and the journal has a policy of publishing individual component articles as soon as they become accepted for publication. If you have proposals or ideas for a Special Issue, I invite you to write directly to the journal offices, who will bring forward the proposal for prompt appraisal by the editorial board.

In terms of in-house management, the journal office in Wuhan continues to work efficiently on behalf of the scientific community. I am confident in stating that I feel that *Vision* is administratively in good hands for its next phase of development.


Andrew J. Parker 15 December 2019


